# Development and Application of a Multiplex PCR Assay for Simultaneous Detection of Tomato Yellow Leaf Curl Virus and Tomato Leaf Curl New Delhi Virus

**DOI:** 10.3390/v17030322

**Published:** 2025-02-27

**Authors:** Hongxia Hu, Jie Zhang, Xiaoyin Wu, Li Li, Yajuan Qian

**Affiliations:** 1 Institute of Biotechnology, College of Agriculture and Biotechnology, Zhejiang University, Hangzhou 310058, China; 22216069@zju.edu.cn (H.H.); 22316261@zju.edu.cn (J.Z.); 22416282@zju.edu.cn (X.W.); 2 Key Laboratory of Agro-Products Postharvest Handling, Ministry of Agriculture and Rural Affairs, College of Biosystems Engineering and Food Science, Zhejiang University, Hangzhou 310058, China; lili1984@zju.edu.cn

**Keywords:** multiplex PCR, tomato yellow leaf curl virus, tomato leaf curl New Delhi virus, simultaneous detection

## Abstract

Tomato leaf curl New Delhi virus (ToLCNDV) and tomato yellow leaf curl virus (TYLCV) are two important viral pathogens that severely affect *Solanaceae* and *Cucurbitaceae* plants. In order to reduce the further spread of these viruses, it is crucial to establish an efficient and reliable method to accurately detect the viruses. In this study, a multiplex PCR assay for the simultaneous detection of TYLCV and ToLCNDV was established. Three primer pairs designed from conserved regions within the coat protein or movement protein-encoding regions of the respective viruses were employed in the assay. The optimization of parameters such as primer concentration was set at 0.15 μM/0.15 μM, 0.25 μM/0.25 μM, and 0.50 μM/0.50 μM for ToLCNDV-DNA-A-F/R, TYLCV-F/R, and ToLCNDV-DNA-B-F/R primer pairs. At optimal primer concentrations, the multiplex PCR method demonstrates effective performance with an annealing temperature ranging from 51 °C to 66 °C. The specificity of the assay evaluated by testing against other begomoviruses showed no evidence of cross-amplification. Further sensitivity analysis performed using a serially diluted plasmid containing viral targets as templates demonstrated high sensitivity with a detection limit of 10^3^ copies/μL. Field surveys utilizing the multiplex PCR assay successfully identified the infection of TYLCV and ToLCNDV in field-collected samples.

## 1. Introduction

ToLCNDV is a member of the *Begomovirus* genus in the *Geminiviridae* family with a bipartite genome consisting of DNA-A and DNA-B [1,2]. DNA-A contains seven open reading frames (ORFs) encoding five distinct proteins: AC1 (Replication initiator protein, Rep), AC2 (Transcriptional activator protein, TrAP), AC3 (Replication enhancer protein, Ren), AC4, AC5, AV1 (coat protein, CP), and AV2. The DNA-B component comprises two ORFs, BC1 and BV1, which encode movement protein (MP) and nuclear shuttle protein (NSP), respectively. ToLCNDV was first identified in tomato in New Delhi, India in 1995 [3], and has since been reported in various regions including China [4]. ToLCNDV is transmitted by the whitefly in a persistent manner and produces typical symptoms including leaf curling, yellowing, leaf chlorotic patches, stunted growth, and fruit deformation, mainly in infected *Solanaceae* and *Cucurbitaceae* plants [5,6,7,8,9,10,11]. The rapid dissemination of ToLCNDV is threatening the production of a range of crops across multiple countries [9,12].

TYLCV, which belongs to a monopartite begomovirus containing only a DNA-A component, is one of the most prominent viral pathogens affecting tomato crops [13,14]. The disease caused by TYLCV, known as tomato yellow leaf curl disease (TYLCD), was first reported in the Jordan Valley, Israel, in the 1950s [15]. In China, TYLCV was first confirmed to infect tomato in 2006 [16]. Beyond tomato, TYLCV can extensively infect a variety of economically important crops, including pumpkin, bean, pepper, cucurbit, and eustoma [17,18,19,20,21]. TYLCV-infected tomato plants exhibit severe symptoms such as stunted growth, small yellow misshapen leaves that usually curl upward, and yellow erect shoots [22,23,24]. Due to its highly invasive nature and the lack of sufficient control measures to limit its dissemination, TYLCV is spreading on a global scale and leading to a significant decline in crop yield and substantial economic losses [14,16,25,26].

The implementation of rapid, sensitive, specific, and cost-effective detection methods for viral diseases is of paramount importance for epidemiological surveillance and the establishment of effective disease management practices [27,28]. Currently, several available methods are employed for the detection of begomoviruses, including ELISA, polymerase chain reaction (PCR), Rolling Circle Amplification (RCA), Loop-Mediated Isothermal Amplification (LAMP), and Recombinase-Mediated Isothermal Amplification (RPA/RAA) [28,29,30,31,32]. ELISA detection of viruses relies on high-quality and specific monoclonal antibodies, which are often time-consuming to develop, and their sensitivity can generally vary depending on the target virus species [33,34]. LAMP and RPA/RAA offer advantages in terms of simplicity and portability, which do not require advanced instruments and are usually visual. LAMP detection exhibits high sensitivity due to the use of several primers, and Caruso et al. developed a real-time LAMP assay for the detection of ToLCNDV [31]. However, LAMP generates multimeric amplification products, which can limit downstream applications of its products, such as direct sequencing, cloning, and restriction analysis, compared to PCR [35]. Similarly, RPA/RAA has gained attention for its rapid amplification and operational convenience. Zhou et al. developed a recombinase polymerase amplification-lateral flow dipstick assay for the detection of TYLCV [23]. Despite these advantages, RPA/RAA faces certain limitations and challenges. The potential for primer mismatches in similar DNA sequences can usually result in false positive results by RPA [36].

Molecular diagnostic tools for plant DNA viruses primarily rely on PCR-based methods. Recently, a multiplex PCR detection assay has been developed [37]. Multiplex PCR enables the simultaneous detection of multiple viruses by incorporating multiple pairs of primers and amplifying several target templates within a single reaction system [38]. Due to its high specificity and sensitivity, this method provides a relatively cost-effective, fast, and reliable method to simultaneously identify several plant viruses in a large number of samples [39]. For example, multiplex PCR has been successfully employed to differentiate between TYLCV and TYLCV-Mld clades, as well as to detect complex infections involving four begomoviruses in cassava [40,41]. However, it is worth noting that the sensitivity of multiplex PCR can be influenced by the number of targets being amplified. Additionally, challenges such as primer-primer interaction and varying optimal amplification conditions for different templates highlight the need for careful screening and optimization of specific primers and reaction conditions to ensure reliable and accurate results [42].

Given that plants infected by TYLCV or ToLCNDV often exhibit similar symptoms in their hosts, and considering the frequent need for simultaneous detection of these two viruses, we developed and optimized a multiplex PCR assay for the rapid, sensitive, and specific detection of TYLCV and ToLCNDV. Our results demonstrated this system can be highly specific for the target viruses by testing against other begomoviruses and is capable of simultaneously detecting TYLCV and ToLCNDV infections in field-collected samples.

## 2. Materials and Methods

### 2.1. Virus and Plant Material

#### 2.1.1. Virus Resources

Virus-positive leaf samples containing the following viruses: tomato leaf curl China virus (ToLCCNV), tomato leaf curl Taiwan virus (ToLCTWV), tomato golden mosaic virus (TGMV), ramie mosaic virus (RAMV), tomato yellow leaf curl Yunnan virus (TYLCYnV), tobacco curly shoot virus (TbCSV), tomato yellow leaf curl China virus (TYLCCNV), ToLCNDV, and TYLCV were identified by our laboratory and stored in −80 °C condition.

#### 2.1.2. Field Plant Sampling Showing Viral Symptoms

Ten field samples exhibiting symptoms of yellowing and curling phenotypes in the leaves were collected from October 2023 to April 2024 in Zhejiang Province, China. Specific information about field samples is shown in Table S1. Upon collection, the samples were quick-frozen in liquid nitrogen and subsequently stored at −80 °C conditions for preservation.

### 2.2. DNA Isolation

The total DNA from the leaves was extracted using FastPure^®^ Plant DNA Isolation Mini Kit (Vazyme, Nanjing, China) according to the producer’s instructions. The quality and concentration of the extracted DNA were evaluated using a spectrophotometer (Nanodrop, Thermo Fisher Scientific, Waltham, MA, USA). The DNA was stored at −20 °C and served as a template for subsequent multiplex PCR assay.

### 2.3. Primer Used for Multiplex PCR Assay

Primers for the multiplex PCR assay were designed based on sequence alignments of conserved regions within CP and MP encoding regions of ToLCNDV-DNA-A and ToLCNDV-DNA-B, as well as the CP encoding regions of TYLCV (Table 1). All the primer sets were synthesized by a commercial provider (Tsingke, Beijing, China).

### 2.4. Construction of Recombinant Plasmids Containing the Target DNA for Analysis of the Sensitivity of Multiplex PCR Assay

The fragments derived from ToLCNDV CP (768 bp), ToLCNDV MP (837 bp), or TYLCV CP (733 bp) were amplified through PCR using 2× TOROBlue^®^ Flash KOD Dye Mix (TOBOYO, Shanghai, China). The primer pairs for amplification are shown in Table S2. The above-resulting fragments were inserted into the pCE3 vector using the Ultra-Universal TOPO Cloning Kit (Vazyme, Nanjing, China) to generate pCE3-ToLCNDV-CP, pCE3-ToLCNDV-MP, and pCE3-TYLCV-CP and subsequently sequenced, respectively.

### 2.5. Optimization of Multiplex PCR Assay

To establish optimal amplification conditions for the target sequences in the multiplex PCR assay, two critical PCR-related parameters are evaluated: the annealing temperatures and the concentrations of primers. Viral DNA extracted from infected plants or plasmid DNA containing the target sequences was used as the template, while double-distilled water (ddH_2_O) served as the negative control. The multiplex PCR reaction system was performed using the 2×Rapid Taq Master Mix (Vazyme, Nanjing, China).

The optimization of primer concentration was initially conducted. Three primer pairs targeting ToLCNDV DNA-A, TYLCV, and ToLCNDV DNA-B (each at a stock concentration of 10 μM) were tested in varying amounts and proportions, with final concentrations of 0.25 μM/0.25 μM/0.25 μM, 0.25 μM/0.25 μM/0.30 μM, 0.25 μM/0.25 μM/0.40 μM, 0.25 μM/0.25 μM/0.50 μM, 0.20 μM/0.25 μM/0.50 μM, 0.15 μM/0.25 μM/0.50 μM, 0.10 μM/0.25 μM/0.50 μM, and 0.05 μM/0.25 μM/0.50 μM. Following the determination of the optimal primer concentration ratio, six annealing temperature gradients—51, 54, 57, 60, 63, and 66 °C—were tested to identify the ideal annealing temperature.

### 2.6. Sensitivity and Specificity of Multiplex PCR Assay

The sensitivity of the multiplex PCR assay was evaluated using different concentrations of plasmids containing the target viral sequences. The pCE3-ToLCNDV-CP, pCE3-ToLCNDV-MP, and pCE3-TYLCV-CP plasmids were sequentially diluted with ddH₂O to concentrations ranging from 10^6^ to 10^1^ copies/μL, respectively. To determine the sensitivity of the assay, equal amounts of each plasmid with the same concentration were mixed and used as templates in the multiplex PCR reactions. A negative control (ddH_2_O) was included in all PCR experiments to ensure the specificity of the assay.

The specificity of this multiplex PCR assay was evaluated using DNA samples containing nine distinct begomoviruses, respectively, including ToLCCNV, ToLCTWV, TGMV, RAMV, TYLCYnV, TbCSV, TYLCCNV, ToLCNDV, and TYLCV as templates to assess the assay’s ability to specifically detect ToLCNDV and TYLCV without cross-reactivity with other begomoviruses.

### 2.7. Validation of Multiplex PCR Assay in Field Samples

According to the optimized conditions of multiplex PCR, total plant DNA extracted from field-collected samples showing virus-like symptoms was subjected to detect virus infection. The DNA extracted from healthy tomato plants served as a negative control to ensure the reliability and specificity of the detection process.

## 3. Results

### 3.1. Development of Multiplex PCR Assay to Simultaneously Detect TYLCV and ToLCNDV

#### 3.1.1. Development of Multiplex PCR Assay

**Figure 1:**
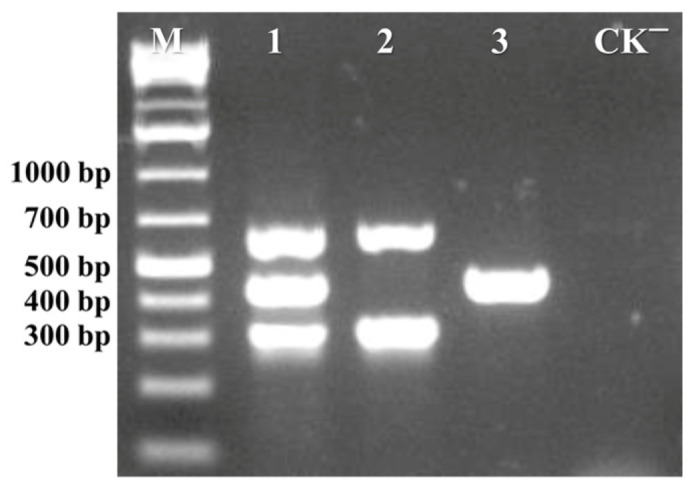
Establishment of multiplex PCR assay. Lane 1 represented the amplification products with three primer pairs (ToLCNDV-DNA-A-F/R, ToLCNDV-DNA-B-F/R, and TYLCV-F/R) with a DNA mixture containing both ToLCNDV and TYLCV as the template. Lane 2 represented the amplification products using ToLCNDV-DNA-A-F/R and ToLCNDV-DNA-B-F/R primer pairs with DNA containing only ToLCNDV as the template. Lane 3 represented the amplification products using TYLCV-F/R primer pairs using DNA containing only TYLCV as a template. Lane M corresponded to the GeneRuler 1 kb Plus DNA Ladder, and Lane CK^−^ represented the negative control.

Virus-specific primers for the detection and differentiation of ToLCNDV and TYLCV were individually designed based on conserved regions within the CP genes of ToLCNDV and TYLCV, as well as the MP gene of ToLCNDV. The initial validation of primer pairs was evaluated using mixtures of virus-positive DNA as templates. The multiplex PCR results demonstrated that all three pairs of primers were capable of amplifying respective target sequences of ToLCNDV and TYLCV in a single reaction (Figure 1).

#### 3.1.2. Primer Concentration Optimization of Multiplex PCR Assay

**Figure 2:**
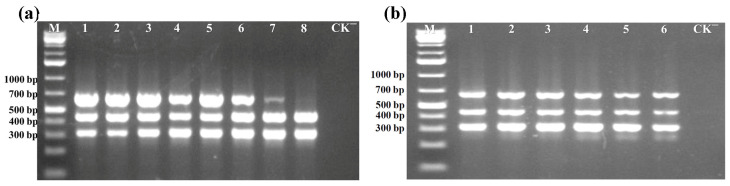
Optimization of multiplex PCR assay. (**a**) Optimization of primer concentrations. Lanes 1–8 represented multiplex PCR reactions performed with varying final concentrations of the three primer pairs (ToLCNDV-DNA-A-F/R, TYLCV-F/R, and ToLCNDV-DNA-B-F/R) as follows: 0.25 μM/0.25 μM, 0.25 μM/0.25 μM and 0.25 μM/0.25 μM; 0.25 μM/0.25 μM, 0.25 μM/0.25 μM and 0.30 μM/0.30 μM; 0.25 μM/0.25 μM, 0.25 μM/0.25 μM and 0.40 μM/0.40 μM; 0.25 μM/0.25 μM, 0.25 μM/0.25 μM and 0.50 μM/0.50 μM; 0.20 μM/0.20 μM, 0.25 μM/0.25 μM and 0.50 μM/0.50 μM; 0.15 μM/0.15 μM, 0.25 μM/0.25 μM and 0.50 μM/0.50 μM; 0.10 μM/0.10 μM, 0.25 μM/0.25 μM and 0.50 μM/0.50 μM; 0.05 μM/0.05 μM, 0.25 μM/0.25 μM and 0.50 μM/0.50 μM; (**b**) Optimization of annealing temperatures. Lanes 1–6 represent reactions performed at annealing temperatures of 51 °C, 54 °C, 57 °C, 60 °C, 63 °C, 66 °C, respectively. Lane M represents the GeneRuler 1 kb Plus DNA Ladder. Lane CK^−^ corresponded to the negative control.

Following the determination of the primers, the step was to optimize the primer concentration for multiplex PCR to achieve balanced and efficient amplification of all target sequences. The three primer pairs were tested at varying concentrations in the multiplex PCR reactions. The brightness of the target amplification bands, as visualized on agarose gel electrophoresis, was analyzed to determine the optimal primer concentrations. The results indicated that the amplification bands specific for ToLCNDV-DNA-B gradually increased in intensity as the concentration of ToLCNDV-DNA-B-F/R primers was incrementally raised (Figure 2a). Based on these observations, the optimal concentrations of ToLCNDV-DNA-A-F/R, TYLCV-F/R, and ToLCNDV-DNA-B-F/R primers carried out in the multiplex PCR were determined as follows: 0.15 μM/0.15 μM for ToLCNDV-DNA-A-F/R, 0.25 μM/0.25 μM for TYLCV-F/R, and 0.50 μM/0.50 μM forToLCNDV-DNA-B-F/R, respectively.

#### 3.1.3. Annealing Temperature Optimization of Multiplex PCR Assay

After determining the optimized concentration for three primer pairs, a gradient of annealing temperature ranging from 51 °C to 66 °C was tested in multiplex PCR. Analysis of the specific amplification bands using agarose gel electrophoresis revealed that all three pairs of primers worked well in this temperature range, with no major difference in effect (Figure 2b). To facilitate the subsequent experimental verification, the annealing temperature for the subsequent experimental conditions was set to 57 °C. Therefore, the thermal cycling conditions for simultaneous detection of TYLCV and ToLCNDV were established as follows: 1 cycle at 95 °C for 3 min; 32 cycles at 95 °C for 15 s, 57 °C for 15 s, and 72 °C for 15 s; and a final extension at 72 °C for 5 min.

### 3.2. Sensitivity and Specificity Detection of Multiplex PCR Assay

#### 3.2.1. Sensitivity Detection

**Figure 3:**
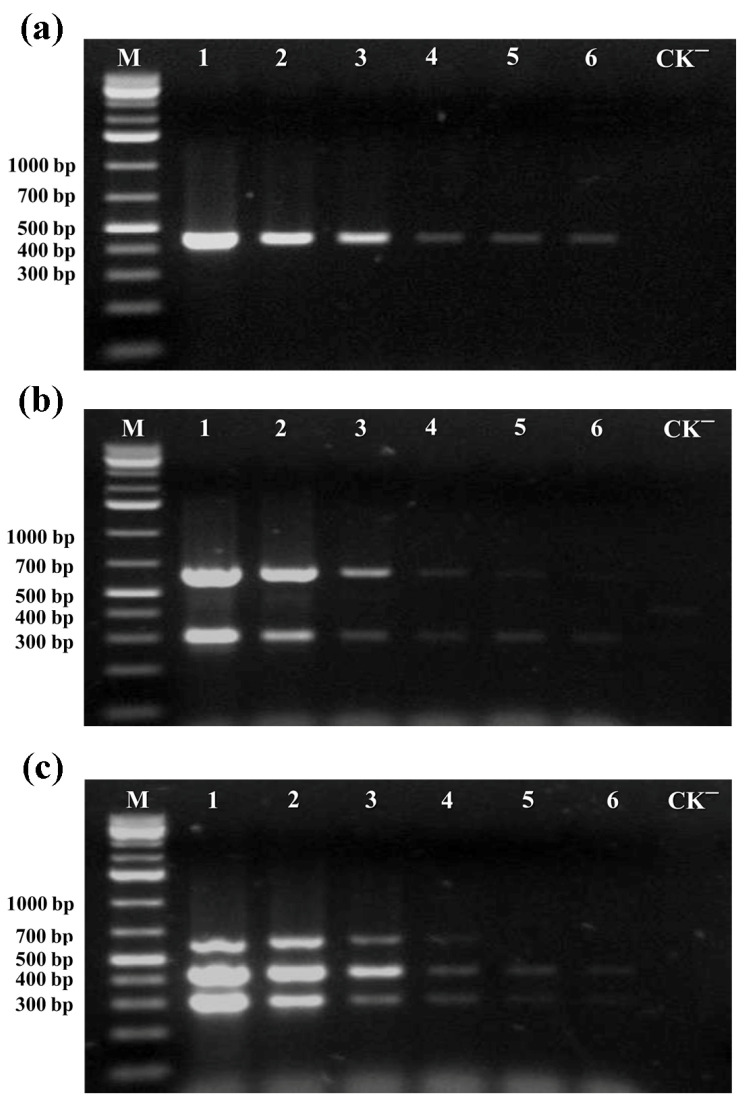
Sensitivity detection of the multiplex PCR assay was evaluated using serial dilutions of the target plasmid. (**a**) Sensitivity of a single PCR assay specific for TYLCV was evaluated; (**b**) Sensitivity of the multiplex PCR specific for ToLCNDV was evaluated; (**c**) Sensitivity of the multiplex PCR specific for ToLCNDV and TYLCV was evaluated. In all assays, lanes 1–7 represented reactions performed with target plasmid concentrations of 10^6^, 10^5^, 10^4^, 10^3^, 10^2^, and 10^1^ copies/μL, respectively. Lane M corresponded to the GeneRuler 1 kb Plus DNA Ladder, and Lane CK^−^ represented the negative control.

To evaluate the sensitivity of the multiplex PCR assay, a plasmid containing the target sequences of TYLCV, ToLCNDV DNA-A, or ToLCNDV DNA-B was serially diluted to concentrations ranging from 10^6^ copies/μL to 10^1^ copies/μL, respectively, and served as templates for PCR amplification. The sensitivity for the individual detection of TYLCV or ToLCNDV, using either the TYLCV CP plasmid or an equal mixture of the ToLCNDV CP and MP plasmids as templates, was 10^1^ copies/μL and 10^3^ copies/μL (Figure 3a,b). As shown in Figure 3c, the results revealed that the detection limit for the simultaneous detection of TYLCV and ToLCNDV was 10^3^ copies/μL when a mixture of positive targets containing equal concentrations of the CP and MP plasmids as templates. Taken together, these results confirmed the high detection sensitivity of the multiplex PCR assay for simultaneous detection of TYLCV and ToLCNDV.

#### 3.2.2. Specificity Detection

**Figure 4:**
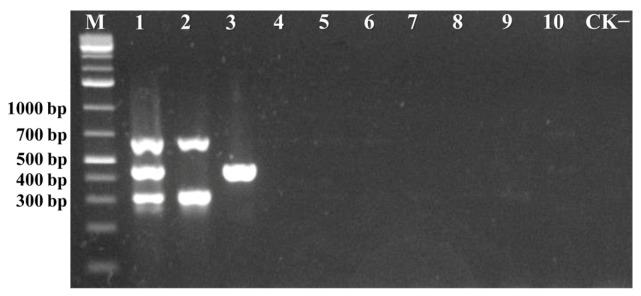
The specificity of the multiplex PCR assay for the detection of TYLCV and ToLCNDV was analyzed. Lane 1 represented a positive sample containing a mixture of ToLCNDV and TYLCV. Lane 2 represented a positive sample containing only ToLCNDV. Lane 3 represented a positive sample containing only TYLCV. Lanes 4–10 represented positive samples containing ToLCCNV, TGMV, RAMV, TYLCCNV, TYLCYnV, ToLCTWV, or TbCSV, respectively. Lane M represented the GeneRuler 1 kb Plus DNA Ladder, and Lane CK^−^ corresponded to the negative control.

To assess the specificity of the multiplex PCR assay, DNA samples containing various begomoviruses such as ToLCCNV, ToLCTWV, TGMV, RAMV, TYLCYnV, TbCSV, TYLCCNV, ToLCNDV, and TYLCV were tested for the presence of ToLCNDV and TYLCV. Following amplification under the optimized multiplex PCR conditions, the results demonstrated that the assay can efficiently and precisely detect ToLCNDV and TYLCV without producing non-specific amplification products from these DNA samples (Figure 4). These findings confirm that the multiplex PCR assay exhibits high specificity and is suitable for the simultaneous and accurate detection of TYLCV and ToLCNDV.

### 3.3. Validation of the Multiplex PCR Assay on Field Plant Samples

**Figure 5:**
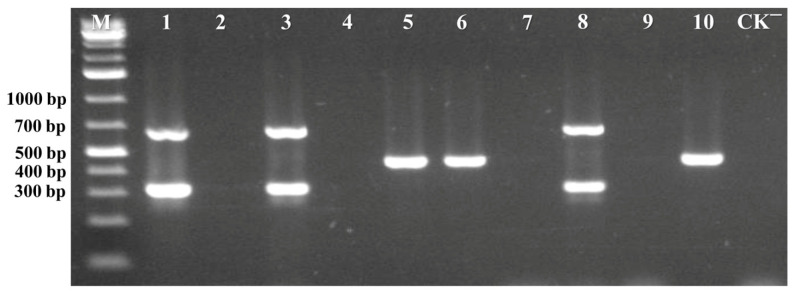
The multiplex PCR assay in field-collected plant samples. Lanes 1 and 3 represented samples from cucumber. Lane 2 represented samples from watermelon. Lanes 4–10 represented samples from tomato. Lane M represented the GeneRuler 1 kb Plus DNA Ladder, and Lane CK^−^ corresponded to the negative control.

The optimal multiplex PCR system was applied to survey the infection of TYLCV and ToLCNDV in ten field-collected samples from cucumber, watermelon, and tomato plants. The results revealed that three samples tested positive for ToLCNDV, and three samples tested positive for TYLCV (Figure 5). These findings demonstrate the utility and reliability of the newly developed multiplex PCR method for the accurate and effective detection of TYLCV and ToLCNDV in field-collected plant samples.

## 4. Discussion

TYLCV and ToLCNDV are both classified within the genus *Begomovirus* of the *Geminiviridae* family. These viruses are emerging pathogens that are widely distributed in tropical, subtropical, and temperate regions of the world and cause severe damage to numerous economically important crops, particularly those in the *Solanaceae* and *Cucurbitaceae* families [43]. Effective management of these viruses is critical to minimizing agricultural losses and ensuring food security [44].

Practical, economical, effective, and sensitive virus detection methods are essential for the prevention and control of plant viruses. In recent years, numerous assays have been developed for the detection of TYLCV and ToLCNDV. For instance, Caruso et al. developed an in-field real-time LAMP assay for the detection of ToLCNDV [31]. Similarly, Xie et al. successfully established a dot enzyme-linked immunosorbent assay (dot-ELISA) and direct tissue blot immunoassay, which have been effectively used to detect TYLCV in field plants and insect vectors [29]. Despite the advantages of serological methods, such as the ability to process a large number of samples in a relatively short period, they are encumbered by certain limitations. These include reduced sensitivity, the requirement for multiple steps, and the inability to accurately detect more than one virus in a single reaction. In contrast, molecular diagnostic tools, such as PCR, usually offer higher sensitivity and specificity. However, single PCR is limited to the individual amplification of each virus, making it more resource-intensive and costly, especially when multiple viruses need to be detected. Multiplex PCR, on the other hand, enables the simultaneous detection of multiple viruses in a single reaction, making it a more efficient and cost-effective approach. This technique is particularly valuable for identifying mixed infections or viruses with highly similar genome sequences. In this study, we demonstrate that a multiplex PCR assay is both specific and sensitive in simultaneously detecting TYLCV and ToLCNDV. This highlights its potential as a critical tool for the simultaneous identification of multiple viruses, thereby enhancing the efficiency of plant virus diagnostics and management.

The development of a reliable multiplex PCR assay requires meticulous optimization of various parameters to ensure accurate and specific detection of target pathogens. The key challenge in multiplex PCR lies in achieving optimal performance of all primer pairs under identical reaction conditions, particularly when detecting multiple targets simultaneously [45]. In this study, we systematically addressed this challenge through a comprehensive optimization process: We conducted extensive experiments to determine the optimal primer concentrations for both TYLCV and ToLCNDV detection. The concentration of each primer pair was individually varied while maintaining fixed concentrations of other components. This approach allowed us to identify the concentration range that produced consistent and balanced amplification of both targets. The annealing temperature is a critical factor influencing primer specificity and amplification efficiency in multiplex PCR. Through gradient PCR experiments, we identified that annealing temperatures in the range of 51–66 °C resulted in efficient and specific amplification of the two target viruses by all three primer pairs with no non-specific binding. To validate the specificity of our newly developed assay, we conducted rigorous testing against a panel of related viruses: The specificity panel included seven closely related begomoviruses: ToLCCNV, ToLCTWV, TGMV, RAMV, TYLCYnV, TbCSV, and TYLCCNV. The assay demonstrated exceptional specificity, showing no cross-reactivity or false-positive amplification with any of the tested viruses. This high specificity is particularly significant given the sequence similarities among begomoviruses, which often pose challenges for virus-specific detection. The successful application of our optimized multiplex PCR system to field-collected samples of cucurbitaceous and solanaceous plants has yielded significant results regarding the detection of TYLCV and ToLCNDV and the results underscore the practical utility and reliability of our newly developed multiplex PCR method in accurately identifying these two begomoviruses in real-world agricultural samples.

The successful optimization of our multiplex PCR assay offers several important advantages: The ability to simultaneously detect TYLCV and ToLCNDV in a single reaction significantly enhances diagnostic throughput and reduces time and resource requirements. The optimal primer concentrations and carefully selected annealing temperature ensure consistent and reproducible results, crucial for accurate disease diagnosis and management. The high specificity of our assay makes it particularly valuable for field applications, where mixed infections with related begomoviruses are common.

## 5. Conclusions

In this study, we established an optimized multiplex PCR assay for the concurrent detection of TYLCV and ToLCNDV. This innovative diagnostic approach demonstrates superior specificity and sensitivity while maintaining practical applicability for field use. Compared to conventional singleplex PCR methods, the developed protocol significantly reduces both processing time and reagent costs without compromising detection accuracy. The implementation of this dual detection system enhances early diagnosis capabilities for TYLCV and ToLCNDV, enabling timely intervention through targeted disease management strategies. This technical advancement supports sustainable agricultural practices by mitigating yield losses caused by viral infections, ultimately contributing to improved crop productivity and economic returns for growers.

## Figures and Tables

**Table 1:** The virus-specific primers used in the multiplex PCR assay.

Viruses	Primer Name	Sequences (5′–3′)	Size (bp)
ToLCNDV-DNA-A	ToLCNDV-DNA-A-F	CTATGGAGCTCGTGCAGTTGTC	651 bp
	ToLCNDV-DNA-A-R	GTAGCATATACAGGATTTGATGCGTGAG	
TYLCV	TYLCV-F	AATCATTTCCACGCCCGTCTC	442 bp
	TYLCV-R	TCCAAAATCCATTGGGCTGTTTCC	
ToLCNDV-DNA-B	ToLCNDV-DNA-B-F	CGTTCGGACCTATAGCCAGATAGG	305 bp
	ToLCNDV-DNA-B-R	CTTCTCTCTCAAGGATAAGAATCCTTGG	

## Data Availability

The data presented in this study are available in this manuscript.

## Supplementary Materials

The following supporting information can be downloaded at: https://www.mdpi.com/article/10.3390/v17030322/s1, Table S1: Information about field samples; Table S2: The primers for amplification of viral functional regions.

## References

[1] Frascati F., Rotunno S., Accotto G.P., Noris E., Vaira A.M., Miozzi L. (2024). Exogenous application of dsRNA for protection against tomato leaf curl New Delhi virus. Viruses.

[2] Prasad A., Prasad M. (2022). Interaction of ToLCNDV TrAP with SlATG8f marks it susceptible to degradation by autophagy. Cell. Mol. Life Sci..

[3] Padidam M., Beachy R.N., Fauquet C.M. (1995). Tomato leaf curl geminivirus from India has a bipartite genome and coat protein is not essential for infectivity. J. Gen. Virol..

[4] Mei Y., Cai L., Wang Y., Li F., Yang X., Yang J., Zhou X. (2023). Molecular characterization and pathogenicity of an infectious clone of tomato leaf curl New Delhi virus isolate infecting *Cucumis melo*. Stress Biol..

[5] Panno S., Iacono G., Davino M., Marchione S., Zappardo V., Bella P., Tomassoli L., Accotto G.P., Davino S. (2016). First report of tomato leaf curl New Delhi virus affecting zucchini squash in an important horticultural area of southern Italy. New Dis. Rep..

[6] Donati L., Bertin S., Gentili A., Luigi M., Taglienti A., Manglli A., Tiberini A., Brasili E., Sciubba F., Pasqua G. (2022). Effects of organic biostimulants added with zeolite on zucchini squash plants infected by tomato leaf curl New Delhi virus. Viruses.

[7] Vo T.T.B., Cho W.K., Jo Y., Lal A., Nattanong B., Qureshi M.A., Tabssum M., Troiano E., Parrella G., Kil E.J. (2023). Transcriptional analysis of the differences between ToLCNDV-India and ToLCNDV-ES leading to contrary symptom development in cucumber. Int. J. Mol. Sci..

[8] Venkataravanappa V., Ashwathappa K.V., Reddy C.N., Shankarappa K.S., Reddy M.K. (2020). Characterization of tomato leaf curl New Delhi virus associated with leaf curl and yellowing disease of watermelon and development of LAMP assay for its detection. 3 Biotech.

[9] Yamamoto H., Wakita Y., Kitaoka T., Fujishiro K., Kesumawati E., Koeda S. (2021). Southeast Asian isolate of the tomato leaf curl New Delhi virus shows higher pathogenicity against tomato and cucurbit crops compared to that of the Mediterranean isolate. Horticult. J..

[10] Bandaranayake W., Wickramarachchi W., Wickramasinghe H., Rajapakshe R., Dissanayake D. (2014). Molecular detection and characterization of begomoviruses associated with cucurbitaceae vegetables in Sri Lanka. J. Natl. Sci. Found. Sri..

[11] Renukadevi P., Devi R.G., Jothika C., Karthikeyan G., Malathi V.G., Balakrishnan N., Rajagopal B., Nakkeeran S., Abd-Allah E.F. (2024). Genomic distinctiveness and recombination in tomato leaf curl New Delhi virus (ToLCNDV-BG) isolates infecting bitter gourd. 3 Biotech.

[12] Sáez C., Flores-León A., Montero-Pau J., Sifres A., Dhillon N.P.S., López C., Picó B. (2022). RNA-Seq transcriptome analysis provides candidate genes for resistance to tomato leaf curl New Delhi virus in melon. Front. Plant Sci..

[13] Wang S., Guo H., Ge F., Sun Y. (2020). Apoptotic neurodegeneration in whitefly promotes the spread of TYLCV. Elife.

[14] Shteinberg M., Mishra R., Anfoka G., Altaleb M., Brotman Y., Moshelion M., Gorovits R., Czosnek H. (2021). Tomato yellow leaf curl virus (TYLCV) promotes plant tolerance to drought. Cells.

[15] Cohen S., Harpaz I. (1964). Periodic, rather than continual acquisition of a new tomato virus by its vector, the tobacco whitefly (*Bemisia tabaci gennadius*). Entomol. Exp. Appl..

[16] Li F., Qiao R., Yang X., Gong P., Zhou X. (2022). Occurrence, distribution, and management of tomato yellow leaf curl virus in China. Phytopathol. Res..

[17] Kil E.J., Kim S., Lee Y.J., Byun H.S., Park J., Seo H., Kim C.S., Shim J.K., Lee J.H., Kim J.K. (2016). Tomato yellow leaf curl virus (TYLCV-IL): A seed-transmissible geminivirus in tomatoes. Sci. Rep..

[18] Moriones E., Navas-Castillo J. (2000). Tomato yellow leaf curl virus, an emerging virus complex causing epidemics worldwide. Virus Res..

[19] Cohen J., Gera A. (1995). Lisianthus leaf curl-a new disease of lisianthus caused by tomato yellow leaf curl virus. Plant Dis..

[20] Navas-Castillo J., Sánchez-Campos S., Díaz J.A., Sáez-Alonso E., Moriones E. (1999). Tomato yellow leaf curl virus-Is causes a novel disease of common bean and severe epidemics in tomato in Spain. Plant Dis..

[21] Reina J., Morilla G., Bejarano E.R., Rodríguez M.D., Janssen D. (1999). First report of *Capsicum annuum* plants infected by tomato yellow leaf curl virus. Plant Dis..

[22] Anfoka G., Haj Ahmad F., Abhary M., Hussein A. (2009). Detection and molecular characterization of viruses associated with tomato yellow leaf curl disease in cucurbit crops in Jordan. Plant Pathol..

[23] Zhou Y., Zheng H., Jiang D., Liu M., Zhang W., Yan J. (2022). A rapid detection of tomato yellow leaf curl virus using recombinase polymerase amplification-lateral flow dipstick assay. Lett. Appl. Microbiol..

[24] Kil E.J., Park J., Choi E.Y., Byun H., Lee K., An C.G., Lee J., Lee G., Choi H.S., Kim C.S. (2018). Seed transmission of tomato yellow leaf curl virus in sweet pepper (*Capsicum annuum*). Eur. J. Plant Pathol..

[25] Akbar A., Al Hashash H., Al-Ali E. (2024). Tomato yellow leaf curl virus (TYLCV) in Kuwait and global analysis of the population structure and evolutionary pattern of TYLCV. Virol. J..

[26] Prasad A., Sharma N., Hari-Gowthem G., Muthamilarasan M., Prasad M. (2020). Tomato yellow leaf curl virus: Impact, challenges, and management. Trends Plant Sci..

[27] Martinelli F., Scalenghe R., Davino S., Panno S., Scuderi G., Ruisi P., Villa P., Stroppiana D., Boschetti M., Goulart L.R. (2015). Advanced methods of plant disease detection. A review. Agron. Sustain. Dev..

[28] Jaybhaye S.G., Chavhan R.L., Hinge V.R., Deshmukh A.S., Kadam U.S. (2024). CRISPR-Cas assisted diagnostics of plant viruses and challenges. Virology.

[29] Xie Y., Jiao X., Zhou X., Liu H., Ni Y., Wu J. (2013). Highly sensitive serological methods for detecting tomato yellow leaf curl virus in tomato plants and whiteflies. Virol. J..

[30] Londoño M.A., Harmon C.L., Polston J.E. (2016). Evaluation of recombinase polymerase amplification for detection of begomoviruses by plant diagnostic clinics. Virol. J..

[31] Caruso A.G., Ragona A., Bertacca S., Montoya M.A.M., Panno S., Davino S. (2023). Development of an in-field real-time LAMP assay for rapid detection of tomato leaf curl New Delhi virus. Plants.

[32] Bang B., Lee J., Kim S., Park J., Nguyen T.T., Seo Y.S. (2014). A Rapid and Efficient Method for Construction of an Infectious Clone of Tomato yellow leaf curl virus. Plant Pathol. J..

[33] Sun A., Wang L., Zhang Y., Yang X., Su Y., Wu X. (2024). Development and application of a duplex RT-RPA assay for the simultaneous detection of cymbidium mosaic virus and odontoglossum ringspot virus. Viruses.

[34] Wang T., Yang J. (2019). Visual DNA diagnosis of tomato yellow leaf curl virus with integrated recombinase polymerase amplification and a gold-nanoparticle probe. Sci. Rep..

[35] Bakheit M.A., Torra D., Palomino L.A., Thekisoe O.M., Mbati P.A., Ongerth J., Karanis P. (2008). Sensitive and specific detection of Cryptosporidium species in PCR-negative samples by loop-mediated isothermal DNA amplification and confirmation of generated LAMP products by sequencing. Vet. Parasitol..

[36] Venbrux M., Crauwels S., Rediers H. (2023). Current and emerging trends in techniques for plant pathogen detection. Front. Plant Sci..

[37] Pallás V., Sánchez-Navarro J.A., James D. (2018). Recent advances on the multiplex molecular detection of plant viruses and viroids. Front. Microbiol..

[38] Devi O.P., Sharma S.K., Sanatombi K., Devi K.S., Pathaw N., Roy S.S., Chanu N.T., Sanabam R., Devi H.C., Singh A.R. (2022). A simplified multiplex PCR assay for simultaneous detection of six viruses infecting diverse Chilli species in India and its application in field diagnosis. Pathogens.

[39] Lafrance R., Valdez-Torres J.B., Villicaña C., García-Estrada R.S., Esparza-Araiza M.J., León-Félix J. (2023). Response surface methodology for optimization of multiplex-PCR protocols for detection of TYLCV, TSWV and *Fol* molecular markers: Analytical performance evaluation. Genes.

[40] Lefeuvre P., Hoareau M., Delatte H., Reynaud B., Lett J.M. (2007). A multiplex PCR method discriminating between the TYLCV and TYLCV-Mld clades of tomato yellow leaf curl virus. J. Virol. Methods.

[41] Aloyce R.C., Tairo F., Sseruwagi P., Rey M.E., Ndunguru J. (2013). A single-tube duplex and multiplex PCR for simultaneous detection of four cassava mosaic begomovirus species in cassava plants. J. Virol. Methods.

[42] Xue B., Shang J., Yang J., Zhang L., Du J., Yu L., Yang W., Naeem M. (2021). Development of a multiplex RT-PCR assay for the detection of soybean mosaic virus, bean common mosaic virus and cucumber mosaic virus in field samples of soybean. J. Virol. Methods.

[43] Fortes I.M., Sánchez-Campos S., Fiallo-Olivé E., Díaz-Pendón J.A., Navas-Castillo J., Moriones E. (2016). A novel strain of tomato leaf curl New Delhi virus has spread to the mediterranean basin. Viruses.

[44] Marchant W.G., Brown J.K., Gautam S., Ghosh S., Simmons A.M., Srinivasan R. (2024). Non-feeding transmission modes of the tomato yellow leaf curl virus by the whitefly *Bemisia tabaci* do not contribute to reoccurring leaf curl outbreaks in tomato. Insects.

[45] Kim I.R., Lim H.S., Choi W., Kang D.I., Lee S.Y., Lee J.R. (2020). Monitoring living modified canola using an efficient multiplex PCR assay in natural environments in south Korea. Appl. Sci..

