# Supplementary material for: Development and Application of a Multiplex PCR Assay for Simultaneous Detection of Tomato Yellow Leaf Curl Virus and Tomato Leaf Curl New Delhi Virus
Source: Viruses. 2025 Feb 27;17(3):322. doi: 10.3390/v17030322 (PMC11945608; doi:10.3390/v17030322)
Supplement: Supplementary file 1 [file viruses-17-00322-s001.zip › viruses-3466156-supplementary.pdf]

Table S1 Specific information on field samples showing virus symptoms

Sample Number	Host	Area
1	Cucumber	Wenzhou, Zhejiang Province
2	Watermelon	Jiaxing, Zhejiang Province
3	Cucumber	Wenzhou, Zhejiang Province
4	Tomato	Jiaxing, Zhejiang Province
5	Tomato	Jiaxing, Zhejiang Province
6	Tomato	Wenzhou, Zhejiang Province
7	Tomato	Wenzhou, Zhejiang Province
8	Tomato	Wenzhou, Zhejiang Province
9	Tomato	Wenzhou, Zhejiang Province
10	Tomato	Wenzhou, Zhejiang Province

Table S2 The primers for amplification of viral functional regions and full-length viruses.

Viruses	Primer Name	Sequences (5′–3′)	Region	Size (bp)
ToLCNDV-DNA-A	ToLCNDV-CP-F	ATGGCGAAGCGACCAGCA	CP	768 bp
	ToLCNDV-CP-R	ATTTGTGACCGAATCATAAAAGTAGATCCG		
TYLCV	TYLCV-CP-F	ATGTCGAAGCGACCAGGC	CP	733 bp
	TYLCV-CP-R	TTGCATACACTGGATTAGAGGCATG		
ToLCNDV-DNA-B	ToLCNDV-MP-F	TTACACGCTCTTCGTTATTGGAGC	MP	837 bp
	ToLCNDV-MP-R	GGAAATGATGGTATGGCTATGGGAG		
